# Real-time high-resolution heterodyne-based measurements of spectral dynamics in fibre lasers

**DOI:** 10.1038/srep23152

**Published:** 2016-03-17

**Authors:** Srikanth Sugavanam, Simon Fabbri, Son Thai Le, Ivan Lobach, Sergey Kablukov, Serge Khorev, Dmitry Churkin

**Affiliations:** 1 Aston Institute of Photonic Technologies, Aston University, Birmingham, B4 7ET, United Kingdom; 2 Institute of Automation and Electrometry SB RAS, 1 Ac. Koptyug Ave., Novosibirsk, 630090, Russia; 3 Zecotek Photonics, Inc., 1120-21331 Gordon Way, Richmond, BC V6W 1J9, Canada; 4 Novosibirsk State University, 630090, Novosibirsk, Russia.

## Abstract

Conventional tools for measurement of laser spectra (*e.g.* optical spectrum analysers) capture data averaged over a considerable time period. However, the generation spectrum of many laser types may involve spectral dynamics whose relatively fast time scale is determined by their cavity round trip period, calling for instrumentation featuring both high temporal and spectral resolution. Such real-time spectral characterisation becomes particularly challenging if the laser pulses are long, or they have continuous or quasi-continuous wave radiation components. Here we combine optical heterodyning with a technique of spatio-temporal intensity measurements that allows the characterisation of such complex sources. Fast, round-trip-resolved spectral dynamics of cavity-based systems in real-time are obtained, with temporal resolution of one cavity round trip and frequency resolution defined by its inverse (85 ns and 24 MHz respectively are demonstrated). We also show how under certain conditions for quasi-continuous wave sources, the spectral resolution could be further increased by a factor of 100 by direct extraction of phase information from the heterodyned dynamics or by using double time scales within the spectrogram approach.

Spectroscopy is an efficient tool in study of many phenomena in bio-photonics, chemistry, communications, non-linear science, and many other areas, including, most notably, laser physics where optical spectrometers are routinely employed for making highly precise measurements in the frequency domain. Apart from the requirement of high spectral resolution, the dynamic character of these phenomena also requires the ability to track their evolution in time. Conventional high-resolution spectroscopy relies in these cases on well-developed mechanically scanned or imaging techniques^1^. However, the achievable scanning rate (or frame rate) is usually too slow, often by many orders of magnitude, to resolve fast spectral dynamics of laser systems. In fibre lasers, the generation spectrum may exhibit significant variation from one cavity round-trip to another and even within one cavity pass^2^. The temporal scale of spectrum evolution may vary from nanoseconds (corresponding to the sampling frequency of hundreds of MHz) in few-metres-long lasers based on ytterbium- or erbium-doped fibres^2^,^3^, to several microseconds (corresponding to the sampling frequency of hundreds of kHz) in typical Raman fibre lasers^4^ or long and ultra-long mode-locked lasers^4^,^5^,^6^,^7^ having cavity length of hundreds of meters to kilometres. Fast spectral dynamics is also observed in delayed-feedback laser systems^8^ and pulsed quantum-cascade lasers with self-scanning of output wavelength due to thermal effects^9^. Spectrometers based on scanned Fabry-Pérot interferometers are not capable of operating faster than few kHz^10^, and detector arrays used in other types of spectrometers do not generally exceed ~20 kHz^11^.

One of the relatively new and actively developed techniques capable of greatly exceeding these limits is the Dispersive Fourier Transform (DFT) approach (see, *e.g.* ref. 12 and references therein). This technique relies on the group velocity dispersion of an optical medium in order to map the spectrum of an optical pulse into the temporal domain. The DFT technique has been advantageously applied to study the generation spectrum dynamics of pulsed fibre lasers^13^,^14^, super-continuum generation^15^, and optical parametric amplifiers^16^. However, this approach imposes its own restrictions on the analysed signal. Since it relies on temporal pulse stretching, the input pulse repetition rate cannot exceed the inverse of the output pulse duration to avoid waveform overlap, thus imposing an upper limit on the simultaneous product of the signal bandwidth, repetition rate, and total system dispersion. Additionally, there is an effective upper limit on the duration of pulses that can be analysed with DFT arising from practically available dispersive media. Although extremely useful in analysis of relatively short pulses, DFT cannot be used to study long pulses, in systems where pulses and continuous waves co-exist, or quasi-continuous wave (CW) lasers. Recently, a time lens parametric spectral analyser based on four-wave mixing has been proposed and demonstrated^17^,^18^. It can be used to characterise spectral dynamics in various sources, however it has limited applicability due to the complexity of the experimental system and limitation to the wavelength resolution of only 0.03 nm, *i.e.* of the GHz order of magnitude.

In this work, we propose a simple and elegant method to measure spectral dynamics of fibre lasers comprising of such continuous and quasi-continuous wave components, by combining the method of time-domain optical heterodyne detection^19^,^20^,^21^ with a specific technique of real-time spatio-temporal measurement^14^,^22^,^23^,^24^,^25^,^26^. We show how by the introduction of a second time scale (here, the cavity round-trip time) and more importantly, utilizing the phase domain information encoded in the heterodyne measurement, it becomes possible to arrive at a frequency resolution that is orders of magnitude higher than those offered by conventional OSAs and even Fourier-transform-based, time-domain approaches. Our proposed method complements existing techniques like the DFT, by allowing the real-time spectral characterisation of long (ns) pulses or CW/quasi-CW radiation.

## Materials and Methods

### Concept of real-time measurements of spectral dynamics in cavity-based systems

**Figure 1:**
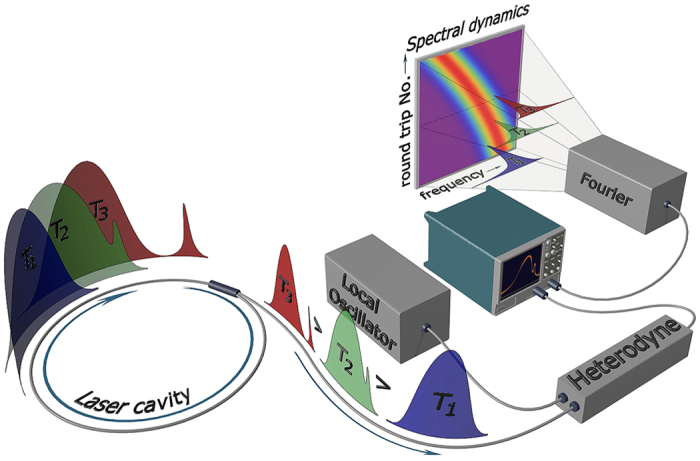
Concept of real-time heterodyne-based measurements of spectral dynamics in fibre lasers. Because of cyclic nature of radiation in the cavity, the output radiation consists of an intensity pattern with a repetition period equal to the cavity round-trip time. Evolution of the intensity pattern, *I*(*t*), over cavity round-trips (evolution time, *T*_*N*_) could be represented as the spatio-temporal intensity dynamics, *I*(*t*, *T*_*N*_). To access the spectral information and measure the spectrum evolution over many cavity round-trips, the spectrum is mapped into time domain via heterodyning and then the heterodyned time trace *I*_*H*_(*t*) is directly measured by a real-time oscilloscope. Further, the trace is processed as a spatio-temporal heterodyned intensity dynamics, *I*_*H*_(*t*, *T*_*N*_). Finally, windowed fast Fourier transform is applied over time coordinate *t*, resulting in measurements of spectral dynamics over cavity round-trips, *I*_*H*_(*ω*, *T*_*N*_). The mathematical background of this procedure is described in Supplementary Materials.

**Figure 2:**
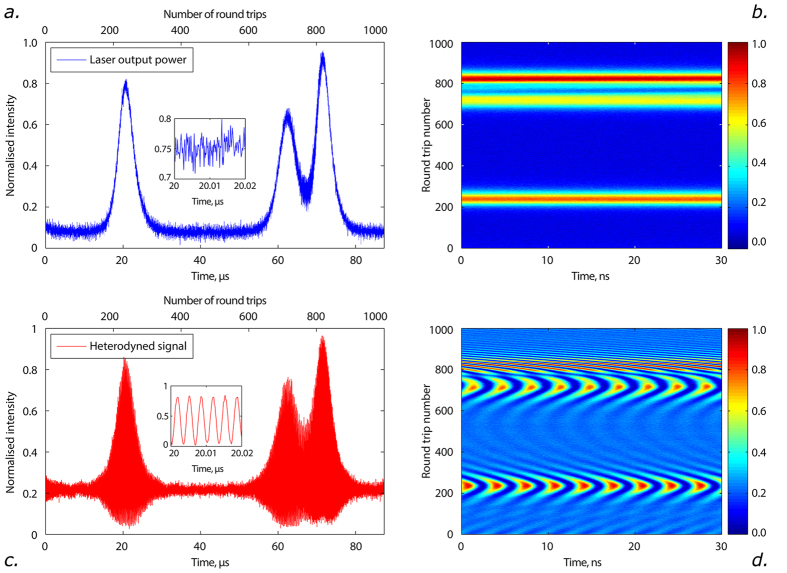
Real-time heterodyned intensity spatio-temporal dynamics. (**a**) Output intensity dynamics, *I*(*t*). Inset: high-resolution details showing that the radiation is quasi-CW on the temporal scale of the round-trip time. (**b**) Spatio-temporal intensity dynamics, *I*(*t*, *T*_*N*_). (**c**) Heterodyned intensity dynamics, *I*_*H*_(*t*), with radio-frequency modulation (inset). (**d**) Spatio-temporal intensity dynamics of the heterodyned intensity, *I*_*H*_(*t*, *T*_*N*_). Phase evolution is clearly visible within and between the pulses.

We illustrate our concept of real-time high-resolution measurements of spectrum evolution on the example of a single-frequency fibre laser, in which the optical spectrum is self-scanned over a broad wavelength range due to the formation of dynamical phase and gain gratings induced by spatial hole burning in population inversion of the active medium^27^. The details of our experimental setup are described in the Supplementary materials. The radiation under study is trapped within the laser cavity making round-trips in it, Fig. 1 (for simplicity shown for a pulsed laser, but also valid for any type of radiation). The intensity dynamics observed at the output of the laser reflects periodic evolution of the radiation in its cavity: the train of output pulses at *T*_1_, *T*_2_, *T*_3_ is actually associated with the same pulse inside the cavity coupled out and sampled at different time instants *T*_*N*_, where *T*_*N*_ = *N* × *τ*_rt_, with *N* denoting numbers of successive cavity round-trips. As a result, the spatio-temporal laser intensity dynamics can be measured in any source^22^, including CW ones. The technique represents an advanced realisation of stroboscopic-type triggered measurements of intensity dynamics of pulsed sources^14^,^23^,^24^,^25^,^26^. In the particular case of the laser under study, generated pulses have duration of ~10 μs (Fig. 2a), being thus much longer than the cavity round-trip time *τ*_rt_ = 85 ns. Therefore, the laser radiation is effectively quasi-CW on the time scale of the round-trip time, see Fig. 2a (inset). The spatio-temporal intensity evolution is trivial in our case, Fig. 2b, indicating that generated broad pulses are stable over evolution time.

To acquire spectral information, we use the well-established heterodyning technique (see, for example, ref. 28). In this technique, the output of a laser under test is linearly mixed with an external source of stable single-frequency radiation, also called the local oscillator. Power-law-based detection of this mixed signal in the time domain is equivalent to a convolution operation in the frequency domain. If the linewidth of the local oscillator is sufficiently narrower than the signal under test, the convolution operation effectively reproduces the spectrum in the radio-frequency spectral domain. Usually, mixed heterodyned signals are measured with conventional radio-frequency (RF) spectrum analysers which convert the heterodyned time-domain intensity dynamics, *I*_*H*_(*t*), into the frequency-domain, *I*(*ω*). However this method cannot resolve fast processes and averages them out because electronic spectrum analysis is limited by relatively slow acquisition rates (see, for instance, ref. 9).

To circumvent this problem, we combine the spatio-temporal methodology^23^ with the specific approach of Brunner *et al.*^19^, where heterodyne detection is now performed in the time domain, Fig. 1. The spectral information is then stored in the heterodyned intensity dynamics in the form of radio-frequency modulation (see Fig. 2c, inset). Further, we take advantage of the natural time scale offered by the cavity round-trip time and represent the heterodyned time-trace *I*_*H*_(*t*), Fig. 2c, as a two-dimensional spatio-temporal dynamics of the heterodyned signal *I*_*H*_(*t*, *T*_*N*_) shown in Fig. 2d. Note that spatio-temporal dynamics of the heterodyned signal also contains information about the spectral composition of the analysed signal, as can be seen from frequency beat bands shifting over the time, Fig. 2d.

**Figure 3:**
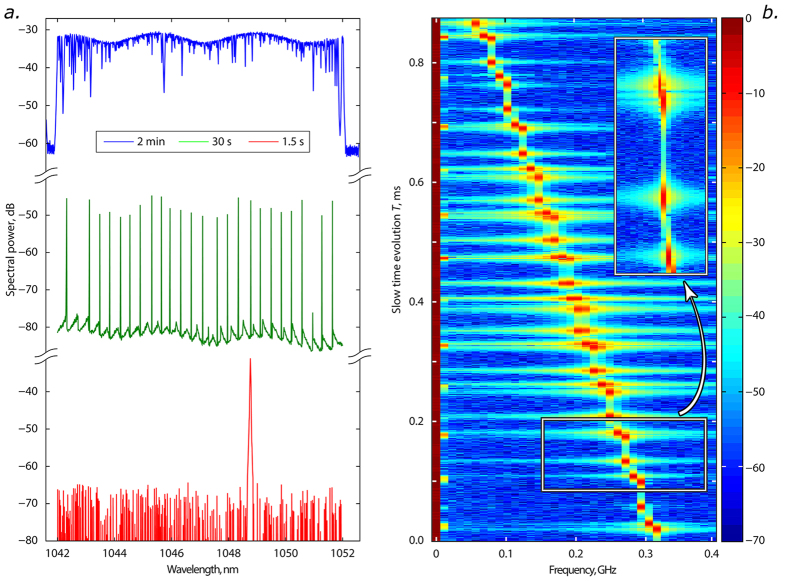
Real-time spectrum dynamics. (**a**) Optical spectrum measured with conventional optical spectrum analyser at different wavelength sweep rates (the data are offset over intensity axes for different sweep times). (**b**) Real-time spectrum dynamics, *I*(*ω*, *T*_*N*_) revealing the self-sweeping behaviour of the fibre laser. The spectral peaks directly correspond to the intensity-domain microsecond-order pulsations. The frequency is defined as an offset frequency from the external local oscillator’s frequency (inset: magnified detail).

To access the spectral information, we now apply a fast Fourier transform (FFT) over time *t*, using the same natural scale unit, *i.e.* the round-trip time, for the window size. As a result, the evolution of instantaneous optical spectrum over cavity round-trips *I*(*ω*, *T*_*N*_) is measured, as illustrated in Fig. 3 (in which linear frequency *f* = *ω*/2*π* is used). The optical spectra are reproduced at the heterodyne beat frequencies, given by the difference between the instantaneous optical frequencies of the signal and the local oscillator. Resolution over the evolution time coordinate in our method is one cavity round trip and its inverse defines the resolution of spectral features. Here, we retain information about evolution over cavity round trips by using non-overlapping windows, which is a natural extension of the spatio-temporal dynamics approach. The technique developed in the present work results in *time-aligned* spectral characterisation of laser radiation, allowing the measurement of spectral dynamics over slow evolution time (round-trips) in a way very similar to those common in the numerical modelling where radiation spectrograms proved to be of extreme importance in understanding of underlying physics^24^,^29^.

As a comparison, we measure the spectrum of the same laser source under study by using both a conventional optical spectrum analyser (OSA), Fig. 3a, and the proposed technique, Fig. 3b. The OSA-based spectral measurement, owing to its limited spectral resolution and the scanned nature of spectral acquisition, is unable to reveal how the spectral characteristics of the source evolve over short time scales. Conversely, the real-time spectral dynamics *I*(*ω*, *T*_*N*_) clearly shows that the laser has quite a narrow spectrum, which evolves continuously over successive cavity round trips, exhibiting self-hopping in the spectral domain, Fig. 3b. Here, the spectral resolution is determined by twice the inverse of the temporal window duration (cavity round-trip time), in this case 24 MHz. The time-domain heterodyne detection methodology clearly outperforms conventional optical spectrum analyzers when one requires high frequency resolution and fast sweep time, as it allows round-trip resolved, high-frequency spectral measurements in quasi-CW fibre lasers. While the maximum optical bandwidth that can be measured by the technique is limited by electrical bandwidth of real-time oscilloscopes and can reach 60 GHz (or twice that number if using multiple oscilloscope channels), it still allows real-time measurements of spectral dynamics in a range over 0.2 nm (0.5 nm) in the 1.0 micron (1.5 micron) range.

## Results and Discussion

### Spectral resolution enhancement via phase reconstruction

The time-domain heterodyne approach for real-time spectral measurement indeed provides a frequency resolution which is at least 3 orders of magnitude higher than in parametric-based approaches^17^,^18^. However, the available resolution is limited by the fundamental Fourier trade-off condition, and is still not high enough to resolve the spectral width of the studied laser, see insert at Fig. 3b. We now extend the above principles of time-domain heterodyne detection, and show how the phase information stored directly in heterodyned intensity spatio-temporal dynamics can be used to improve resolution over frequency.

**Figure 4:**
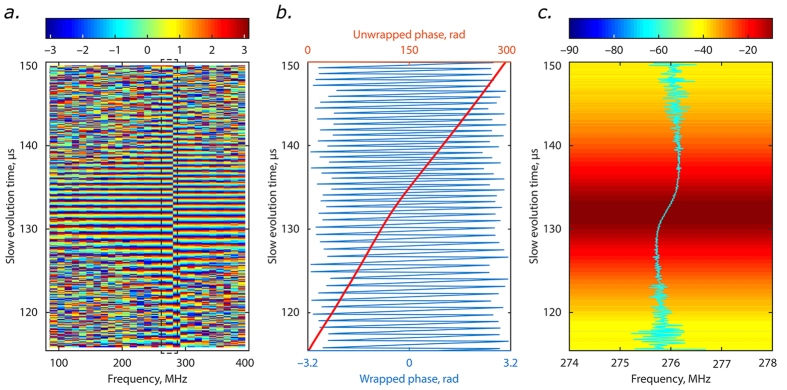
Frequency resolution enhancement via phase reconstruction. (**a**) Evolution of the phase of Fourier components of heterodyned intensity dynamics over frequency and round-trip number. Dashed rectangle highlights the region of interest. (**b**) Phase evolution of the selected Fourier component: blue and green curves denote wrapped and unwrapped phase respectively. (**c**) High-resolution frequency drift within the pulse super-imposed and centered on the initial spectral evolution dynamics of a single mode hopping transition. Note that total frequency range on this graph (4 MHz) which is smaller than initial frequency resolution of 11 MHz.

The heterodyned intensity spatio-temporal dynamics contains precise phase information of the heterodyne carrier frequency, which can be seen as pronounced modulation. Further, it also records the change in the instantaneous frequency, as clearly evidenced by the change in the phase of the modulation over round trips, Fig. 2d. If the studied signal has a narrow spectrum falling below the conventional FFT limit and the intensities of the original (non-mixed) signals remain fairly constant over the window, the argument of Fourier transform directly gives the phase of the carrier (see Supplementary Materials). In essence, this is equivalent to the assumption that the signal can be considered single-frequency over the whole observation period. The phase of the carrier component over successive round trips can then be directly extracted from the experimental heterodyned intensity spatio-temporal evolution (Fig. 4a). Phase unwrapping (addition of π-increments at phase discontinuities) is required as the FFT operation gives values of the phase within the limits [−π, π] (Fig. 4b). The instantaneous frequency evolution can be obtained by simple differentiation of this unwrapped phase over the evolution co-ordinate *T*_*N*_ (Fig. 4c).

With this method, we are able to see that the laser under study exhibits a monotonic chirp within the duration of each long laser pulse (Fig. 4c). The total frequency excursion is less than 1 MHz, which is considerably smaller than the initial FFT-defined spectral resolution of 24 MHz. The effective frequency resolution provided by the phase method is around 100 kHz, which is a two orders of magnitude improvement over the conventional FFT resolution, while the resolution over evolution time is kept equal to 1 round-trip. In this analysis, the same experimental data were used as in Fig. 3b. The frequency resolution depends on the electrical bandwidth (see Eq.13, Supplementary Materials). But even with detection configurations with moderate GHz-order bandwidths, the frequency resolution remains at least an order of magnitude higher than the Fourier window resolution.

Note that the Fourier transform trade-off condition is effectively still in place: while the instantaneous frequency evolution can be tracked with enhanced (up to a factor of 100) frequency resolution, the absolute value of the carrier frequency is still governed by the FFT resolution equalling 24 MHz in our case.

### Spectral resolution enhancement via Wigner-Ville distribution based approach

**Figure 5:**
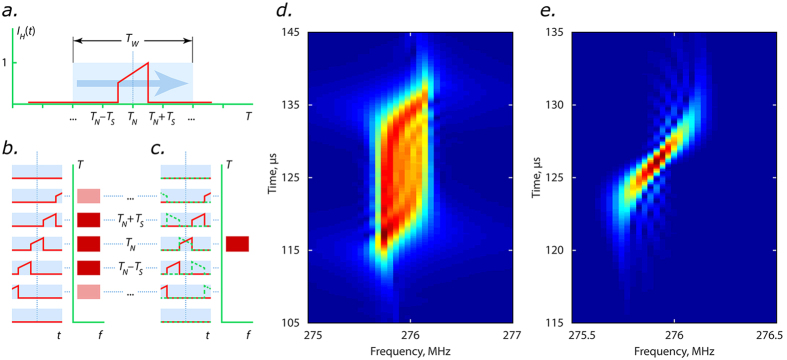
Concept of spectral dynamics restoration by introducing second time scale. The concept is demonstrated on the example of a monochromatic signal located at time instant *T*_N_ with duration of one round-trip. The width *T*_W_ of the window function is chosen to be large enough for the desired frequency resolution. The sliding window time step *T*_S_ is defined by the desired resolution over slow evolution time and should be sufficiently smaller than *T*_W_. (**a**) Monochromatic signal (red curve) and the sliding window function (blue rectangle). (**b**) The product of the signal and window function at different positions of the window moving with step *T*_*S*_ and the corresponding modelled spectrogram. The position of the frequency component can be defined only to the accuracy of *T*_W_. (**c**) Similar illustration for the chosen window function corresponding to the Wigner-Ville distribution. Red curve corresponds to the monochromatic windowed signal, while its time-reversed and time-shifted replica is shown as green dashed curve. Their product is non-zero only at time instant *T*_N_. This results in the position of the frequency component defined with accuracy equal to *T*_S_ ≪ *T*_W_. (**d**) Spectrogram of experimental data calculated as shown in panel (**b**). Spectral dynamics of the same experimental data calculated *via* Eq. (2). High resolution both over frequency and evolution time is achieved. Colour is used to code spectral power density in panels (**d**,**e**).

In this section, we present another approach to increase the spectral resolution, which is not limited to the case of slow varying sinusoid. Extending the temporal window of the Fourier transform beyond the round trip time will improve frequency resolution. However, due to a fundamental trade-off of the Fourier transform between the frequency and temporal resolution, improved frequency resolution will result in coarser resolution over the evolution time coordinate *T*_*N*_ of the spectral dynamics. It is known that spectral dynamics can be restored via a spectrogram approach, *i.e.* by calculating Fourier transform within a moving window^22^. Figure 5d shows the spectrogram of the heterodyned intensity dynamics (experimental data same as in Fig. 4), where we have used a much longer window function *T*_W_ of width 200 round trips to achieve sufficiently high frequency resolution (~120 kHz). The existence of a chirp which was recovered from the phase domain, Fig. 4c, can be still seen in the spectral evolution recovered using spectrogram approach, but the time instant at which a particular frequency component appears can no longer be defined with accuracy higher than the length of the window function *T*_W_. Nevertheless, there still exists essential information in the spectrogram definition, which in principle allows us to retain high resolution over the evolution coordinate while at the same time keeping high resolution over frequency. Indeed, the temporal window in the spectrogram definition is shifted over time at a certain step *T*_S_, in our case equal to one round-trip. Physically, this introduces another temporal scale much smaller than the window size, *T*_S_ ≪ *T*_W_, which could be used to restore the resolution over slow evolution time.

To clarify the concept, let us consider an ideal signal in which some narrowband frequency component is only present for the duration of a single round-trip *N*, *i.e.* this frequency appears at time instant *T*_*N*_ of the evolution co-ordinate, Fig. 5a. Using the window of length *T*_W_ involved in Fourier transform results in the fact that the chosen frequency component will be present in the spectrogram for all time instants, 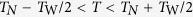, as Fig. 5b demonstrates.

However, if we re-define the window function used in spectrogram definition we can restore the resolution over evolution time. In particular, we use the product of a rectangular profile of width T_W_ and a time-shifted time-reversed copy of the heterodyned signal itself as a window function. That is, we calculate the following function:


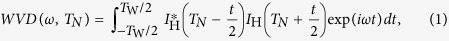


which is essentially the Wigner-Ville Distribution (WVD)^30^ of the heterodyned signal within the window centred around *T*_*N*_. The conjugate term indicates that an analytic extension of the heterodyne time series is used (see Supplementary Materials). The Wigner-Ville distribution was originally proposed in quantum mechanics and it now finds extensive use in real-time process monitoring^31^,^32^, in biomedical applications^33^,^34^, and also for ultrashort pulse characterisation^35^,^36^,^37^.

The introduction of a specific window function results in the fact that the signal and its time-shifted and time-reversed replica overlap only for time exactly equal to *T*_N_, *i.e.* the resulting function is non-zero only for time instant *T*_N_, Fig. 5c. This allows restoration of the position of the frequency component over slow evolution time to the accuracy of the moving window time step *T*_S_. Further, for a signal exhibiting a linear frequency chirp, the WVD gives an unbiased estimate of the instantaneous frequency of the signal, where the frequency resolution is now determined by the width of the window function used (see Supplementary Materials).

Figure 5e shows the spectral dynamics calculated using Eq. 1 for the same region as in Fig. 5d. The resolution over evolution time is considerably improved (by more than an order of magnitude) while high resolution over frequency is retained, Fig. 5e. The resulting frequency evolution shown on Fig. 5e is in excellent agreement with phase extraction procedure presented in Fig. 4c (see Fig. S2 for direct comparison). Note that the resolution over evolution time is not completely restored to the theoretical limit of one round-trip. This is caused by multi-frequency composition (and also non-linear chirp) of the signal. Thus, because of the second time scale external to the Fourier transform procedure and the specific shape of the window function, the spectral dynamics can be tracked simultaneously with a high frequency resolution and high temporal resolution along the evolution co-ordinate *T*.

## Conclusion

In summary, by combining methodologies of time domain-heterodyne detection and spatio-temporal dynamics, we have demonstrated a time-aligned, round-trip time resolved characterisation of spectral dynamics of fibre lasers comprising of continuous and quasi-continuous wave radiation components. In this regard, the methods serve to complement existing real-time spectral measurement techniques for regular pulses like the DFT. We have further shown how by using the Fourier phase domain and WVD approaches, it is possible to obtain frequency resolution of the underlying dynamics that is at least two orders of magnitude higher than that dictated by the Fourier trade-off condition. While the applicability of the phase domain approach is limited to signals satisfying the slowly varying sinusoid approximation, the WVD approach can be extended for the case of more non-trivial higher order frequency excursions^38^. Very recently, a scheme for performing a Fourier-transform based spectral measurement in the optical domain has been proposed, which lifts the restrictions on the nature of the signal interrogated^39^. However, the system has a finite temporal domain response, which results in an averaging of dynamics along the evolution co-ordinate. Indeed, the electrical bandwidth of the detector and oscilloscopes determine the maximum observable optical bandwidth, and currently limit the direct study of broadband optical spectra (around 120 GHz). Nevertheless, the possibility of continuous spectral acquisition over multiple round trip time scales, and the orders of magnitude higher spectral resolutions serve to offer significant advantages for the study of a wide range of lasers. Important examples of systems of interest include long mode-locked lasers with dissipative wave interaction^6^,^23^, Raman lasers^22^,^40^,^41^, and even random lasers^42^, including those based on optical fibres^43^. Even though random lasers do not comprise a cavity of any fixed length, co-existence of localised and extended modes^44^ with different lifetimes may lead to complex spectral dynamics, which is usually lost in conventional spectral measurements. For such systems, the round-trip time can be replaced by an appropriate natural time scale, and the spectral dynamics can then be resolved by the described techniques. The ability to record long time traces (up to 2 billion samples in high-end oscilloscopes) opens the possibility of statistical studies in the spectral domain. Combination of this capability with triggering in temporal domain to focus on spectral content of specific temporal events, for example, rogue ones, can substantially increase the total observation time. Furthermore, spectral pre-filtering of the initial optical signal and simultaneous measurements in different spectral bands can potentially help to reveal internal spectral correlations in real-time, which could be of interest in studies of mode-locking onset in fibre lasers. We anticipate that the demonstrated real-time measurements of spectral dynamics can substantially improve experimental capabilities in study of systems with complex temporal and spectral dynamics.

## Additional Information

**How to cite this article**: Sugavanam, S. *et al.* Real-time high-resolution heterodyne-based measurements of spectral dynamics in fibre lasers. *Sci. Rep.* **6**, 23152; doi: 10.1038/srep23152 (2016).

## Supplementary Material

Supplementary Information
