# Supplementary material for: Real-time high-resolution heterodyne-based measurements of spectral dynamics in fibre lasers
Source: Sci Rep. 2016 Mar 17;6:23152. doi: 10.1038/srep23152 (PMC4794724; doi:10.1038/srep23152)
Supplement: Supplementary Information [file srep23152-s1.pdf]

Supplementary information:
Real-time high-resolution heterodyne-based measurements of spectral dynamics in fibre lasers

Srikanth Sugavanam[1], Simon Fabbri[1], Son Thai Le[1], Ivan Lobach[2], Sergey Kablukov[2], Serge Khorev[3,4], Dmitry Churkin[1,2,4,*]

[1]Aston Institute of Photonic Technologies, Aston University, Birmingham, B4 7ET, United Kingdom,
[2]Institute of Automation and Electrometry SB RAS, 1 Ac. Koptyug Ave., Novosibirsk, 630090, Russia,
[3]Zecotek Photonics, Inc., 1120-21331 Gordon Way, Richmond, BC V6W 1J9, Canada,
[4]Novosibirsk State University, 630090, Novosibirsk, Russia.

1. Optical heterodyne detection in the time domain

1.1. Principle

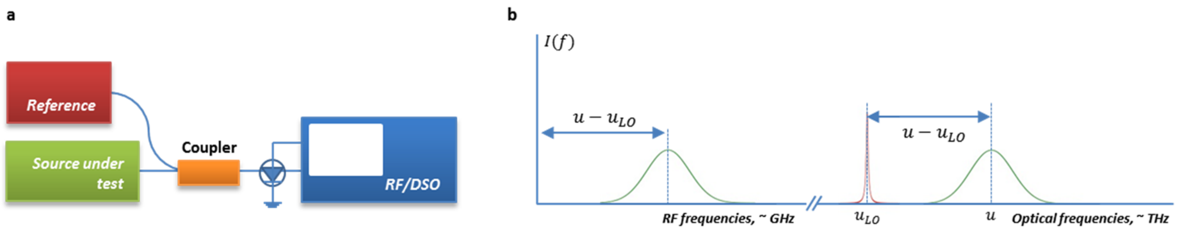

Fig. S1 Principle of optical heterodyne detection in the time domain (a). Schematic experimental layout for optical heterodyne interferometry. Conventionally, a radio frequency power spectrum analyzer (RF) is used for the detection for the heterodyned signal. Here, it is replaced with a digital storage oscilloscope (DSO). (b). Frequency domain representation of heterodyne detection. The optical frequencies u and u_{LO} are beyond the bandwidth of detection of the system, however power law detection results in a convolution between the spectra of the source and the local oscillator. The source spectrum is then reproduced at the beat frequencies $u - u_{LO}$ which fall within the bandwidth of detection.

The time domain heterodyne detection approach[1] can be presented mathematically as follows. If the source under test is of the form

$$E_s(t) = \sqrt{I(t)}.\cos\big(2\pi u(t)t + \Phi(t)\big)$$

Equation 1

and it is linearly mixed with a local oscillator that is characterized by a constant intensity I_{LO}, then the heterodyne signal detected by the photodetector can be given as

$$I_H(t) = I_S(t)/2 + I_{LO}/2 + 2\sqrt{I(t).I_{LO}}\cos[2\pi(u(t) - u_0)t + \Phi(t)]\}$$

Equation 2

where $I_s(t) = E_s(t).E_s^*(t)$, $u(t)$ and u_0 are the instantaneous frequencies of the source and the reference respectively. For clarity, the responsivity term of the photodetector has been dropped. The first two terms are time-averaged components of the direct signals as detected by the bandwidth limited optical detector. The second term form the beat frequencies of interest. An RF spectrum analyser can be used to measure the spectral power

$$P(f) = \langle \int_{-\frac{\Delta f}{2}}^{\frac{\Delta f}{2}} PSD(f).\,df \rangle_{\Delta T}$$

Equation 3

where $PSD(f)$ is the power spectral density, defined as

$$PSD(f) = \frac{1}{\Delta T} \cdot \left| \int_{-\Delta T/2}^{\Delta T/2} I_H(t).\,e^{j2\pi ft} dt \right|^2,$$

Equation 4

Δf is the resolution bandwidth of the RF analyser and ΔT is the acquisition time. Equation 4 is representative of the fact that the RF analyser can only present an averaged picture of the underlying dynamics over the time period ΔT.

For obtaining the real-time spectrum from the time domain heterodyned signal, one can define a spectral function of the form

$$\begin{aligned} S(f,T) &= \int_{-\infty}^{\infty} w(t-T).\,I_H(t) e^{-j2\pi ft} dt \\ &= \int_{-\infty}^{\infty} w(t-T).\,(I(t)/2 + I_{LO}/2) e^{-j2\pi ft} dt \\ &+ 2\int_{-\infty}^{\infty} \sqrt{I(t).\,I_{LO}}\, w(t-T) \cos\big(2\pi\nu(t)t + \Phi(t)\big)\, e^{-j2\pi ft} dt \end{aligned}$$

Equation 5

The defined spectral function is the *windowed Fourier transform* of the heterodyne intensity $I_H(t)$. $w(t)$ is a real and even windowing function centred about an arbitrary time instant T. It signifies the finite duration (i.e. ΔT) of the acquired time domain signal. $\nu(t) = u(t) - u(0)$ is defined as the heterodyne frequency. The first term are the contributions of the direct terms (i.e. baseband terms), arising from a linear superposition of the intensities of the source and the signal. The second term is a mixed product, which is the desired convolution product of interest (i.e. sideband term). If the optical frequency of the local oscillator is so chosen that the difference frequency $\nu(t)$ is much larger than the spectral bandwidth of the source, then the integrals of Equation 5 will be well separated in the frequency domain. The power of the local oscillator is usually chosen to be much larger than that of the source, which further suppresses the contribution of the source in the direct term. Then, the spectrally shifted sideband term gives the dynamic optical spectrum of the source under test measured over a duration equal to the length of the window. From fundamental properties of the Fourier transform, the frequency domain resolution is known to be inversely proportional to the duration of the window ($df \sim 1/\Delta T$). Thus, a larger window can be chosen to improve on spectral resolution. But this comes at the cost of averaging the spectrum over the window duration. Spectral characteristics of laser systems typically evolve at time scales comparable to the cavity round trip time τ_{RT} (~10^2 ns for a cavity of few tens of meters). A Fourier transformed heterodyne signal measured over this time scale would still provide a resolution of sub-picometer (~MHz) order. Thus, time domain heterodyne measurements can be used to recover the spectral evolution in such systems *over consecutive round trips*, while still retaining an appreciable level of spectral resolution. Furthermore, the spectral evolution can be recorded over multitudes of round trips (ten thousand in the present work), only limited by maximal signal length that can be recorded with the oscilloscope.

2. Experimental configuration

The fibre laser used in the experiment exhibits self scanning over a broad wavelength range due to the formation of dynamical phase and gain gratings induced by spatial hole burning in population inversion of the active medium [2]. It has a Fabry-Perot cavity length of 8.5 metres, which corresponds to 85-ns round-trip time. It generates long 10-µs pulses, which are much longer than the cavity round-trip time. The laser generates single-frequency output self-scanned around 1,066 nm. For heterodyning, we used an independent solid-state laser with instantaneous linewidth/phase noise less than 1 kHz measured over a period of 100 milliseconds. The carrier frequency drift of this oscillator was of the order of 1 MHz over a period of 1 minute. The mixed waveform data was recorded with a real-time digital oscilloscope of 2.5 GHz bandwidth.

3. Frequency resolution enhancement using phase domain information

3.1. Principle

Heterodyne detection is a phase sensitive detection technique. This essentially means that if the signal being measured is coherent, the heterodyned signal contains information about the phase evolution of the signal relative to the phase of the local oscillator. If the frequency $u(t)$ of the source under test does not vary with time, its phase would linearly increase with time t. However, if the frequency is not constant, and a derivative of the phase $\Phi(t)$ over time would then give the instantaneous frequency deviations of the laser signal about its mean frequency.

For example, let the output of the laser be represented by

$$E_s(t,T) = \sqrt{I(T)}.\exp\left[i\left(2\pi u(T)t + \Phi(T)\right)\right]$$

Equation 6

Note the use of the variable T for the frequency and phase instead of t, which is to emphasize that the phase changes over cavity round trip time scales τ_{RT}, which is larger than the time scale t. The signal can then be regarded as a slowly varying sinusoid, which offers a significant simplification of Equation 5 as follows. The windowed FFT signal can be expressed as

$$S(f,T) = \int_{-\infty}^{\infty} w(t-T).I_s(t,T).\exp(j2\pi f t)\,dt$$

Equation 7

where the window function is taken to be the rectangular function

$$w(t) = rect\left(\frac{t}{\tau_{RT}}\right) = \begin{cases} 1, & -\tau_{RT}/2 < t < \tau_{RT}/2 \\ 0, & otherwise \end{cases}$$

Equation 8

Equation 7 can be expressed as

$$S(f,T) = \frac{(I(T) + I_{LO})}{2} . \int_{T-\frac{\tau_{RT}}{2}}^{T+\frac{\tau_{RT}}{2}} \exp(-j2\pi f t)\, dt + \sqrt{I(T).I_{LO}} \int_{T-\frac{\tau_{RT}}{2}}^{T+\frac{\tau_{RT}}{2}} \exp\left(j\left(2\pi \nu t + \Phi(T)\right)\right) \exp(-j2\pi f t)\, dt + \sqrt{I(T).I_{LO}} \int_{T-\frac{\tau_{RT}}{2}}^{T+\frac{\tau_{RT}}{2}} \exp\left(-j\left(2\pi \nu t + \Phi(T)\right)\right) \exp(-j2\pi f t)\, dt$$

where, $\nu = u(T) - u_0$, as before. Further, we have,

$$S(f,T) = \frac{(I(T) + I_{LO})}{2} . e^{-j2\pi f T} . \tau_{RT} . \mathrm{sinc}(\pi f \tau_{RT}) + \sqrt{I(T).I_{LO}} . \tau_{RT} . e^{-j2\pi f T} e^{j\Phi(T)} \mathrm{sinc}(\pi(f-\nu)\tau_{RT}) + \sqrt{I(T).I_{LO}} . \tau_{RT} . e^{-j2\pi f T} e^{-j\Phi(T)} \mathrm{sinc}(\pi(f+\nu)\tau_{RT})$$

Equation 9

For $f = \nu$, the above equation takes the form

$$S(\nu,T) = (I(T) + I_{LO})/2 . e^{-j2\pi \nu T} . \tau_{RT} . \mathrm{sinc}(\pi \nu \tau_{RT}) + \sqrt{I(T).I_{LO}} . \tau_{RT} . e^{-j2\pi \nu T} . e^{j\Phi(T)}$$

Equation 10

where the contribution of the third term of Equation 9 (i.e., the negative frequency term) has been neglected. The first term in the above expression can be ignored as long as $\pi \nu \tau_{RT} \gg 1$. In our experiments, the round trip time $\tau_{RT} \sim 100\ ns$ and the heterodyne frequency $\nu \gtrsim 100\ MHz$, therefore $\pi \nu w \gtrsim 10$ for all values of T. Thus, the complex Fourier coefficient at the RF carrier frequency provides direct measurement of the signal phase during a given round trip, which can be calculated as:

$$\Phi(T) = Arg\{S(\nu,T)\} = \Phi(T) + 2\pi \nu T$$

Equation 11

where the second term is a constant. The phase over consecutive round trips can then be obtained by shifting the window. Thus, information of the instantaneous frequency evolution of the signal can be obtained as

$$\delta f(T) = \frac{1}{2\pi} . \frac{\Phi(T_{i+1}) - \Phi(T_i)}{T_{i+1} - T_i} \equiv \nu + \frac{1}{2\pi} . \frac{\Delta\Phi}{\tau_{RT}}$$

Equation 12

where $\Phi(T_i)$ and $\Phi(T_{i+1})$ are the values of the phase obtained from consecutive round trips.

3.2. Spectral resolution in the FFT Phase domain approach

If the temporal resolution of the oscilloscope is δt, then the smallest frequency deviation that can be measured is given by

$$\delta f(T) = \frac{1}{2\pi} . \frac{2\pi \nu \delta t}{\tau_{RT}}$$

Equation 13

where ν is the heterodyne frequency. As in the present case $\delta t = 50\ ps$ and $\tau_{RT} = 80\ ns$, $\delta f(T) = 125\ kHz$. This value is substantially higher than the frequency resolution offered by the windowed Fourier transform. The resolution enhancement in turn arises from the ability of the oscilloscope to measure and resolve minute changes in phase. As revealed by Equation 13 above, higher the sampling

rate, the better the phase resolution. Thus, the introduction of the second time scale allows measurement of phase differences with a resolution determined by the oscilloscope, and hence allows the extraction of the instantaneous frequency deviations with a resolution better by two orders of magnitude compared to the conventional windowed FFT approach.

Note that the above expression measures *deviations* about the mean frequency ν, while the indeterminacy in ν is still governed by the Fourier transform properties. Note that the FFT algorithm in itself does not introduce any phase errors, and thus the ultimate measurement precision is determined by how fast the phase changes over round trips, the resolution of the oscilloscope, the value of the heterodyne frequency ν, and the round trip time period τ_{RT}. With this additional information, one can resolve the sub-MHz frequency shift that is demonstrated by the mode hopping laser as described in the main text (see Fig. 3).

4. Resolution enhancement using Wigner Ville distributions

4.1. Principle

The Wigner Ville Distribution (WVD) of a time varying signal $\boldsymbol{x(t)}$ can be written as

$$WVD_x(f,T) = \int_{-\infty}^{\infty} w(\tau - T).\, x\left(T + \frac{\tau}{2}\right).\, x^*\left(T - \frac{\tau}{2}\right).\exp(-j2\pi f\tau).\, d\tau\,.$$

Equation 14

where the windowing function $w(t)$ reflects finite observation time. The WVD can be envisioned as a windowed Fourier transform operation, where the window function is a time reversed and conjugated replica of the signal itself. As illustrated in the main text (see Fig. 5), the specific choice of the window function leads to a high resolution along the slow time evolution co-ordinate. Specifically, the chosen form of the window function leads to the result that for a signal exhibiting a linear frequency chirp, the WVD leads to an unbiased estimate of the instantaneous frequency [3].

For instance, let $x(t)$ represent a signal with a linear frequency chirp of the form

$$x(t) = A_0.\exp[i(2\pi\nu_0 t + \alpha t^2)]$$

Equation 15

where α is the chirp parameter. The WVD of the above function can be written as

$$WVD(f,T) = \int_{T-\frac{M\tau_{rt}}{2}}^{T+\frac{M\tau_{RT}}{2}} A_0^2.\, e^{i2\pi\nu_0\left(T+\frac{\tau}{2}\right)} e^{i\alpha\left(T+\frac{\tau}{2}\right)^2} e^{-i2\pi\nu_0\left(T-\frac{\tau}{2}\right)} e^{-i\alpha\left(T-\frac{\tau}{2}\right)^2} e^{-i2\pi f\tau} d\tau$$

Equation 16

where the window function is taken to be of a rectangular function of width $M\tau_{RT}$ located about the time instant T. We thus have,

$$WVD(f,T) = A_0^2 \int_{T-\frac{M\tau_{RT}}{2}}^{T+\frac{M\tau_{RT}}{2}} e^{-i2\pi\left[f-2\left(\nu_0+\frac{\alpha}{2\pi}T\right)\right]\tau} d\tau$$

Equation 17

which gives,

$$WVD(f,T) = A_0^2.M\tau_{RT}.e^{-j2\pi\nu_0 T}\mathrm{sinc}\left[\pi\left\{f - 2\left(\nu_0 + \frac{\alpha}{2\pi}T\right)\right\}M\tau_{RT}\right]$$

Equation 18

Thus, the WVD essentially gives the *instantaneous frequency* (IF) of the chirped signal. The windowing operation eliminates all quadratic terms in frequency, leaving behind a Dirac kernel for the windowed Fourier transform operation (Equation 22). This essentially implies that the linearly chirped signal has now been replaced with a single sinusoidal signal, which has a frequency that is twice of the instantaneous frequency of the signal at the centre of the window, and now spans across the whole window. The instantaneous frequency can now be estimated precisely *with **an arbitrarily high resolution*** simply by enlarging the size of the window, i.e. choosing a larger value of M. In this fashion, the WVD can potentially offer a high resolution along both frequency and the evolution co-ordinates for such linearly chirped signals.

4.2. Computing the WVD using the FFT

The form of Equation 14 suggests that the WVD can be calculated using existing FFT algorithms. However, before the straightforward application of the routine, signal conditioning is required. The following steps are to be carried out in order to recover the WVD signal

- **Analytic extension of the signal**

The Fourier frequency spectrum $S(f)$ of a real valued signal $x(t)$ is a function that comprises of both positive and negative frequencies, with the property of Hermitian symmetry:

$$S(-f) = S^*(f)$$

A multiplication operation in the time domain results in convolution products between positive and negative frequencies in the frequency domain, which gives rise to spurious interferences. These spurious artefacts can complicate interpretation when analyzing heterodyned signals. To avoid this, a real valued signal is *analytically extended*. The analytic extension of a real valued signal $\boldsymbol{x(t)}$ is defined as

$$z(t) = x(t) + jy(t),$$

where

$$y(t) = \mathcal{H}\{x(t)\} = (-j\mathrm{sgn}f)S(f)$$

with

$$sgn(f) = \begin{cases} -1, & f < 0 \\ 0, & f = 0 \\ 1, & f > 0 \end{cases}$$

The analytic signal has the property

$$Z(f) = \mathcal{F}\{z(t)\} = 0\,for\,f < 0$$

Thus, the analytic function $z(t)$ can be used in lieu of the real valued signal $x(t)$ to eliminate interference effects.

- **The WVD as the absolute value of the FFT**

The WVD can now be calculated directly using the form of Equation 14 using the standard FFT algorithm. The phase terms that remain arise from the shifting operation of the rectangular window, and also the difference in the definition of integration limits in the discrete Fourier transform. These phase bias terms can be removed by simply considering the absolute value of the FFT calculated.

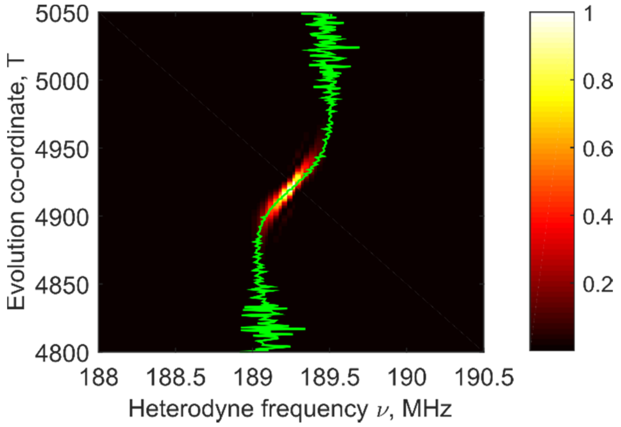

Fig. S2. Comparing the instantaneous frequency evolution as obtained with the WVD and the FFT Phase domain approach (green curve)

Fig. S2 shows the comparison the WVD operation and the FFT phase domain approach. There is good agreement between the two methodologies, particularly near the centre of the profile where the frequency excursion is linear. While the presented form of the WVD is limited to linearly chirped signals, the WVD can also be extended to study polynomial and higher order forms of frequency evolution [4].

4.3. WVD for multicomponent signals

When a time varying signal comprises more than one frequency, it is called a multicomponent signal. The WVD of such a signal results in the generation of cross products that lie in between the two frequencies, owing to the quadratic nature of the Fourier kernel (Equation 22). For example, Fig. S3a shows an overlay of the windowed FFT and the WVD for such a multicomponent signal comprising of two frequencies. The mixed product does not interfere with the originally occurring frequencies, and one can filter away the mixed product.

Fig. S3(b) shows a larger span of the WVD shown in Fig. 4 of the main text in the logarithmic scale. The temporal window size of 200 round trips was chosen such that there remained only one predominant component within the window. In this scenario, mixed products are observed (indicated by the dotted ellipse), however their contribution to the primary frequency of interest can be neglected. Alternatively, this situation can be avoided by using an appropriate smoothing function over the time frequency plane (see for example, [5]), or by decomposing the signal using a suitable procedure (see for example, [6]) before the application of the procedure.

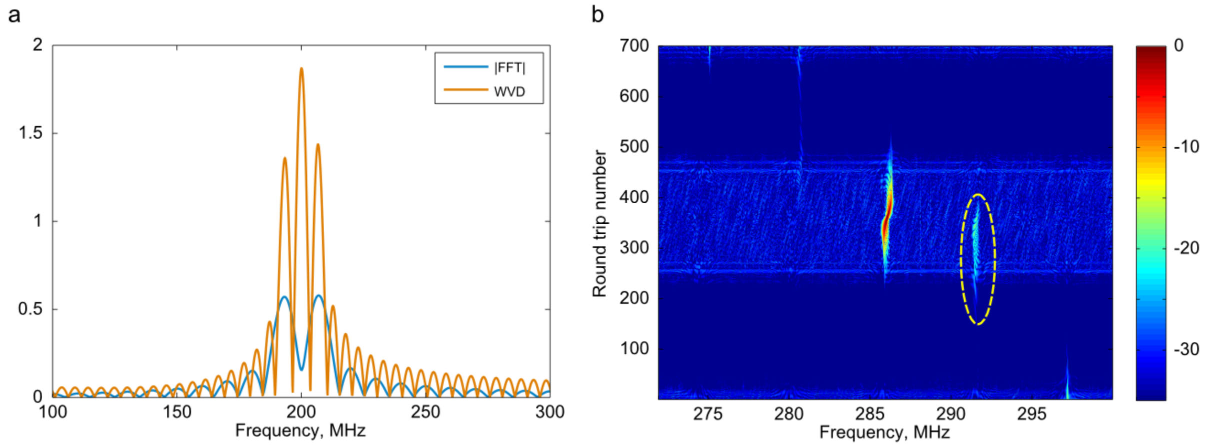

Fig. S3 Cross products in the WVD (a). FFT and WVD for a two-component signal (b). The WVD for the experimental heterodyned signal. The cross product term is indicated by the dotted ellipse. Color scale indicates normalized power in dB.

References

1 Brunner, D., Porte, X., Soriano, M. C. & Fischer, I. Real-time frequency dynamics and high-resolution spectra of a semiconductor laser with delayed feedback. *Scientific reports* **2** (2012).

2 Lobach, I. A., Kablukov, S. I., Podivilov, E. V. & Babin, S. A. Self-scanned single-frequency operation of a fiber laser driven by a self-induced phase grating. *Laser Physics Letters* **11**, 045103 (2014).

3 Boashash, B. *Time frequency analysis*. (Gulf Professional Publishing, 2003).

4 Boashash, B. & O'Shea, P. Polynomial Wigner-Ville distributions and their relationship to time-varying higher order spectra. *Signal Processing, IEEE Transactions on* **42**, 216-220 (1994).

5 Flandrin, P. in *Acoustics, Speech, and Signal Processing, IEEE International Conference on ICASSP '84.* 266-269.

6 Qian, S. & Chen, D. Decomposition of the Wigner-Ville distribution and time-frequency distribution series. *Signal Processing, IEEE Transactions on* **42**, 2836-2842, doi:10.1109/78.324750 (1994).
